# Use of ultrasound-measured internal jugular vein collapsibility index to determine static intracardiac pressures in patients with presumed pulmonary hypertension

**DOI:** 10.1186/s13613-019-0595-7

**Published:** 2019-10-28

**Authors:** Raj Parikh, Matthew Spring, Janice Weinberg, Christine C. Reardon, Harrison W. Farber

**Affiliations:** 1 Division of Pulmonary and Critical Care Medicine, Boston University Medical Center, Boston, MA USA; 2 Department of Internal Medicine, Boston University School of Medicine, Boston, MA USA; 3 Department of Biostatistics, Boston University School of Public Health, Boston, MA USA; 4 Division of Pulmonary, Critical Care and Sleep Medicine, Tufts Medical Center, Boston, MA USA; 5 Boston University Medical Center, 72 East Concord Street, R304, Boston, MA 02118 USA

**Keywords:** Hemodynamics, Heart failure, Pulmonary hypertension, Diagnostic testing, Echocardiography, Imaging, Ultrasound

## Abstract

**Background:**

Bedside ultrasound helps to estimate volume status in critically ill patients and has traditionally relied on diameter, respiratory variation, and collapsibility of the inferior vena cava (IVC) to reflect fluid status. We evaluated collapsibility of the internal jugular vein (IJ) with ultrasound and correlated it with concomitant right heart catheterization (RHC) measurements in patients with presumed pulmonary hypertension.

**Methods and results:**

We studied 71 patients undergoing RHC for evaluation of pulmonary hypertension. Using two-dimensional ultrasound (Sonosite, Washington, USA), we measured the diameter of the IJ at rest, during respiratory variation, and during manual compression. Collapsibility index during respiration (respiratory CI) and during manual compression (compression CI) was calculated. We correlated mean right atrial pressure (mRAP) and pulmonary artery occlusion pressure (PAOP) defined by RHC measurements with respiratory and compression CI. A secondary goal was examining correlations between CI calculations and B-type natriuretic peptide (BNP) levels. Baseline characteristics demonstrated female predominance (*n* = 51; 71.8%), mean age 59.5 years, and BMI 27.3. There were significant correlations between decrease in compression CI and increase in both mRAP (Spearman: − 0.43; *p* value = 0.0002) and PAOP (Spearman: − 0.35; *p* value = 0.0027). In contrast, there was no significant correlation between respiratory CI and either mRAP (Spearman: − 0.14; *p* value = 0.35) or PAOP (Spearman:− 0.12; *p* value = 0.31). We also observed significant negative correlation between compression CI and BNP (Spearman: − 0.31; *p* value = 0.01) but not between respiratory CI and BNP (Spearman: − 0.12; *p* value = 0.35).

**Conclusion:**

Increasing use of ultrasound has led to innovative techniques for estimating volume status. While prior ultrasound studies have used clinical parameters to estimate fluid status, our study used RHC measurements and demonstrated that compression CI potentially reflects directly measured mRAP and PAOP.

## Background

Currently, precise determination of cardiac filling pressures and biventricular volume status requires invasive monitoring with a pulmonary artery catheter. Ultrasonography of the inferior vena cava (IVC) can non-invasively estimate the mean right atrial pressure (mRAP), but it can be misleading and limited, especially among critically ill patients or if visualization of the IVC is suboptimal [1, 2]. Despite these limitations, bedside ultrasound has become a versatile modality for assessing volume status in critically ill patients using measurements such as diameter, variation with respiration, and overall collapsibility of the IVC [3–9]. Recently, ultrasound characterization of the internal jugular vein (IJ), specifically diameter and cross-sectional area, has been shown to correlate with and predict central venous pressure (CVP) [10–15]. Furthermore, these IJ measurements are comparable and at times superior to IVC measurements [16]. However, no studies have correlated IJ ultrasonography with invasively measured hemodynamics. The aim of the current study was to correlate measurements of the IJ by bedside ultrasound with measurements obtained during concomitant right heart catheterization (RHC).

## Methods

### Study design and patient selection

We conducted a prospective study of 71 consecutive patients undergoing RHC as part of routine evaluation for pulmonary hypertension (PH) at Boston University Medical Center, Boston, MA, USA. The study was approved by the Institutional Review Board of Boston University Medical Center and the requirement for informed consent was waived.

### Data extraction: ultrasound measurements

**Fig. 1:**
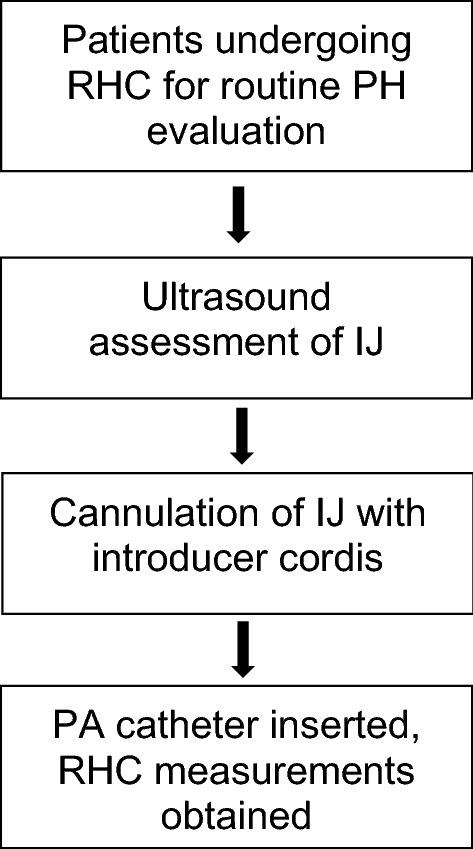
Study Design

Clinical and laboratory data were collected for all patients before and during the procedure. Collected data from the electronic medical record included past medical history and laboratory tests. Procedural data included ultrasound measurements and RHC measurements (Fig. 1).

RHC was performed in a fluoroscopy-equipped catheterization laboratory with patients positioned at the level of their respiratory comfort; 62 (87.3%) participants were supine for the entirety of the procedure. Using a bedside two-dimensional ultrasound (Sonosite, Washington, USA), the diameter of the IJ at rest, during respiratory variation (inspiration and expiration), and during manual compression (measured at 2 cm of depth from the skin) was measured. All ultrasound measurements were completed prior to the RHC. Trained Pulmonary/Critical Care physicians with certification to complete central venous and pulmonary artery catheterizations performed the ultrasound measurements and the subsequent RHC.

**Fig. 2:**
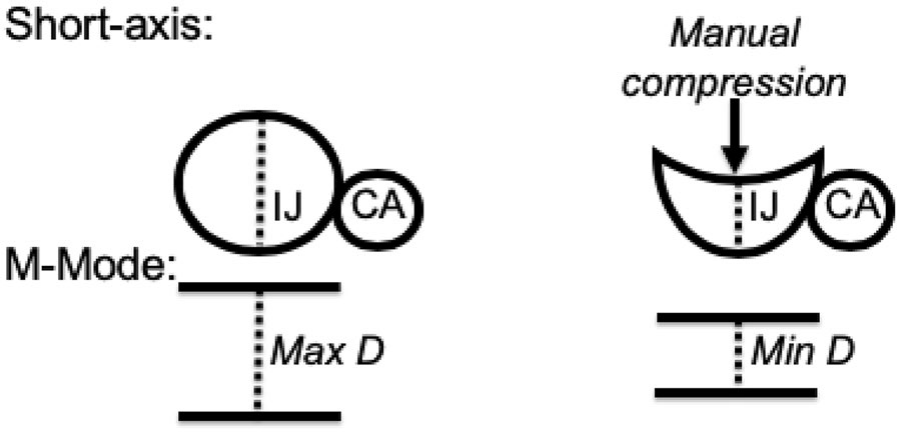
Schematic representation of ultrasound findings in Short-axis and M-mode. 2-cm manual compression of the IJ is noted on the right panel of the figure. *IJ* internal jugular vein, *CA* carotid artery, *Max* maximum, *D* diameter, *Min* minimum

IJ measurements were recorded with the linear transducer probe. The IJ was visualized by placing the ultrasound transducer perpendicular to the skin in the transverse plane at a level just above the clavicle. The IJ was identified by compression as well as by color Doppler imaging and Pulse Wave Doppler. Sufficient ultrasound gel was used to prevent direct skin contact with the transducer, helping to limit the amount of pressure applied and avoid significant influence of external compression on the IJ diameter at rest [7]. Measurements were obtained by an M-mode scan on the ultrasound device. The maximum anterior–posterior diameter of the IJ was measured at rest followed by variations during the respiratory cycle, where the patient was asked to take a deep breath in, breathe out, and then hold their breath at the end of expiration for one second. Subsequently, minimal pressure (measured via ruler at 2 cm of depth from the superficial skin) via the transducer was applied on the IJ to induce extrinsic manual compression (Fig. 2). The antero-posterior diameter of the IJ was then re-measured. Accuracy of the measurements was based on agreement between the operator and the supervising physician (HWF for all patients). The collapsibility index of respiratory variation (respiratory CI) and the collapsibility index of manual extrinsic compression (compression CI) were computed using the following calculation: (maximum diameter − minimum diameter)/maximum diameter.

### Data extraction: RHC measurements

Following the ultrasound measurements, RHC was performed using best practice guidelines established by the European Respiratory Society (ERS) [17]. RHC measurements included right atrial systolic, diastolic, and mean pressures (mRAP); systolic, diastolic, and end diastolic right ventricular pressures (RVSP, RVDP, RVEDP, respectively); systolic, diastolic, and mean pulmonary artery pressures (PASP, PADP, mPAP, respectively); pulmonary artery occlusion pressure (PAOP). Cardiac output by both thermodilution (TD) and Fick methods. Cardiac index, pulmonary vascular resistance (PVR), and systemic vascular resistance (SVR) were calculated in standard fashion.

### Study outcomes

The primary outcome of the study was the correlation between respiratory CI, compression CI, and volume status defined by the RHC measurements. Additionally, we evaluated the correlation between respiratory CI, compression CI, and B-type natriuretic peptide (BNP) levels, measured pre-procedurally.

### Statistical analysis

Summary statistics are reported on all variables including n (%) for categorical and mean ± standard deviation for continuous measures. Spearman correlations were used to quantify the linear relationship between continuous measures. All analyses were performed using SAS v9.4 with *p* < 0.05 considered statistically significant.

## Results

The study included 71 consecutive patients. Baseline characteristics demonstrated female predominance (*n* = 51; 71.8%), mean age of 59.5 years, and a BMI of 27.3 (Table 1). Prominent co-morbidities included congestive heart failure (*n* = 32; 45.1%) and interstitial lung disease (*n* = 30; 42.3%). By RHC, 35 patients (49.3%) were diagnosed with Group 1 PH (pulmonary arterial hypertension; PAH) and 7 patients (9.9%) did not have PH; the remaining 29 patients were diagnosed with Groups 2–5 PH. The mean BNP level within 6 months prior to the RHC was 567.6 pg/mL.

**Table 1:** Baseline characteristics of patients undergoing right heart catheterization and pre-procedural bedside ultrasound

Baseline characteristics	All patients *N* = 71
Female sex	51 (71.8%)
Mean age (SD)	59.5 (12.6)
Mean BMI (SD)	27.3 (7.1)
Mean BNP (SD)	567.6 (1082.6)
*Co*-*morbidities*	
CHF	32 (45.1%)
ILD	30 (42.3%)
OSA	18 (25.4%)
COPD	9 (12.7%)
ESRD	5 (7.0%)
*PH group*	
No PH	7 (9.9%)
Group 1	35 (49.3%)
Group 2	13 (18.3%)
Group 3	7 (9.9%)
Group 4	4 (5.6%)
Group 5	5 (7.0%)

*BMI* body mass index, *BNP* B-type natriuretic peptide, *CHF* congestive heart failure, *ILD* interstitial lung disease, *OSA* obstructive sleep apnea, *COPD* chronic obstructive pulmonary disease, *ESRD* end-stage renal disease, *PH* pulmonary hypertension

Prior to RHC, ultrasound measurements of IJ maximum diameter at rest, during inspiration, and during manual extrinsic compression were obtained (Table 2). IJ measurements of the right (*n* = 68; 95.8%) and left (*n* = 3; 4.2%) sides were obtained. Mean IJ diameter at rest was 1.17 cm, at inspiratory breath hold was 1.39 cm, and 0.73 cm with external compression. Calculated respiratory CI was 16.2% and compression CI was 40.0%.

**Table 2:** Bedside ultrasound measurements of internal jugular vein

Ultrasound measurements	Mean (SD)
IJ at rest (cm)	1.17 (0.41)
IJ at inspiratory breath hold (cm)	1.39 (0.44)
IJ with external compression (cm)	0.73 (0.43)
Respiratory CI (ratio)	0.16 (0.13)
Compression CI (ratio)	0.40 (0.24)

*IJ* internal jugular vein, *CI* collapsibility index

Mean measurements obtained by RHC in this cohort were: mRAP 6.4 mmHg, RVEDP 8.5 mmHg, mPAP 35.3 mmHg, and PAOP 10.0 mmHg (Table 3). The average cardiac output was 5.39 L/min by TD and 5.16 L/min by Fick. The average cardiac index was 3.03 L/min/m2 by TD and 2.86 L/min/m2 by Fick. PVR was 482.3 dynes/sec/cm^−5^ by TD and 512.1 dynes/sec/cm^−5^ by Fick; SVR was 1462.9 dynes/sec/cm^−5^ by TD and 1529.7 dynes/sec/cm^−5^ by Fick.

**Table 3:** Right heart catheterization measurements

RHC measurements	Mean (SD)
mRAP (mmHg)	6.4 (5.4)
RVEDP (mmHg)	8.5 (5.9)
mPAP (mmHg)	35.3 (13.0)
PAOP (mmHg)	10.0 (6.1)
Cardiac output TD (L/min)	5.4 (2.0)
Cardiac output Fick (L/min)	5.2 (1.7)
Cardiac index TD (L/min/m^2^)	3.0 (1.1)
Cardiac index Fick (L/min/m^2^)	2.9 (0.8)
PVR TD (dynes/s/cm^−5^)	482.3 (420.2)
PVR Fick (dynes/s/cm^−5^)	512.1 (448.0)
SVR TD (dynes/s/cm^−5^)	1462.9 (578.0)
SVR Fick (dynes/s/cm^−5^)	1529.7 (621.9)

*mRAP* mean right atrial pressure, *RVEDP* right ventricular end diastolic pressure, *mPAP* mean pulmonary arterial pressure, *PAOP* pulmonary artery occlusion pressure, *TD* thermodilution, *PVR* pulmonary vascular resistance, *SVR* systemic vascular resistance

There were significant negative correlations between compression CI and both mRAP (Spearman: − 0.43; *p* value = 0.0002) and PAOP (Spearman: − 0.35; *p* value = 0.0027) measurements (Table 4). In contrast, there was no significant correlation between respiratory CI and either mRAP (Spearman: − 0.14; *p* value = 0.233) or PAOP (Spearman: − 0.12; *p* value = 0.307). In regards to our secondary goal, there was a significant negative correlation between a compression CI and serum BNP (Spearman: − 0.31; *p* value = 0.015), but not between respiratory CI and BNP (Spearman: − 0.12; *p* value = 0.351). Lastly, IJ diameter at rest correlated significantly with mRAP (Spearman: 0.26; *p* value = 0.029) but not with PAOP (Spearman: 0.14; *p* value = 0.238) or BNP (Spearman: 0.15; *p* value = 0.224).

**Table 4:** Spearman correlation coefficients between ultrasound measurements and markers of volume status

Ultrasound measurements	MRAP	PAOP	BNP
IJ diameter at rest	*r* 0.26	*r* 0.14	*r* 0.15
	*p* value 0.029	*p* value 0.238	*p* value 0.224
Respiratory CI	*r* − 0.14	*r* − 0.12	*r* − 0.12
	*p* value 0.233	*p* value 0.307	*p* value 0.351
Compression CI	*r* − 0.43	*r* − 0.35	*r* − 0.31
	*p* value 0.0002	*p* value 0.0027	*p* value 0.015

*CI* compression index, *mRAP* mean right atrial pressure, *PAOP* pulmonary artery occlusion pressure, *BNP* B-type natriuretic peptide

## Discussion

The increasing use of ultrasound to estimate CVP and fluid responsiveness in acutely and/or chronically ill patients has led to several innovative techniques. While prior ultrasound studies have used clinical parameters to estimate volume status, the current study used concomitant RHC measurements, the gold standard in volume assessment. This study demonstrated that compression CI, a simple ultrasound technique requiring minimal operator experience, accurately reflects directly measured mRAP and PAOP in patients with presumed pulmonary hypertension.

### Compression CI

Assessing the ultrasound-measured compressibility of a vein and using it as a non-invasive marker for fluid status could provide yet another component to critical care ultrasound techniques. Volume status is a central component to manage both critically ill patients in an intensive care unit and chronically ill patients, such as those with underlying renal or cardiac disease [18, 19]. Moreover, the accuracy in estimating volume status is essential to improve patient care and therapeutic approaches. Based on the current observations, compression CI of the IJ, a simple, non-invasive technique that only requires basic ultrasound skills, could be used as a surrogate for CVP.

### IJ vs IVC

Use of the compression CI of the IJ provides a novel approach to non-invasive fluid status assessment; however, is it better or easier than use of the IVC as the target venous structure? IVC collapsibility is a well-documented measurement in critical care ultrasound and volume assessment, although evidence for its accuracy is controversial; moreover, in many situations, the IVC is poorly visualized [1, 2, 16]. Additionally, formal echocardiography uses the IVC diameter and collapsibility with sniff to generate an estimated, yet imprecise, assessment of the mRAP [3]. Nevertheless, the IVC has historically been used as the target venous structure; in particular, size of the IVC and its dynamic change during respiration is used to estimate mRAP [3–9]. The current study suggests that use of the IJ may be an accurate and reliable estimation of mRAP during ultrasonography. Although we did not compare IJ and IVC measurements in the current study, the strong correlation observed between IJ diameter at rest and mRAP suggests the need for future studies comparing both ultrasound methods.

### Limitations

The current study has several limitations. 1) The operators were not blinded to the ultrasound and RHC assessments, which could have influenced the results. 2) The cohort included patients with known or suspected pulmonary vascular disease; none were critically ill patients in an intensive care unit. Unfortunately, given the paucity of pulmonary artery catheters inserted in intensive care units currently, it would be very difficult to perform a similar study in such patients. Likewise, we did not assess fluid responsiveness in these patients. 3) We only evaluated patients undergoing RHC in a catheterization laboratory, leading to issues with extrinsic validity and generalizability. 4) Ultrasound probe compression is a non-formal technique commonly used to differentiate venous and arterial structures when obtaining vascular access [20]. In the current study, we used an extrinsic compression of 2 cm at the level of the patient’s skin. Despite the outlined technique, user error in estimating 2 cm and an inability to directly measure the degree of compression could result in variability. Additionally, variability with patient’s body habitus could also affect the ability to provide adequate compression. Although a standardized process of applying compression has yet to be described in the literature, in this study, one individual supervised all the procedures and measurements to assure a standard protocol among the operators. Further studies that apply the use of a pressure manometer could mitigate this variability; such a pressure manometer has been utilized in other ultrasound-based investigations [21]. In a similar manner, standardizing the maneuver used to obtain the respiratory CI was a challenge as well [22].

Ultrasound measurements of the IJ, specifically compression CI, correlate with invasively measured biventricular hemodynamics in this non-critically ill population of patients with presumed pulmonary hypertension. Thus, compression CI may be a useful tool in the non-invasive estimation of intravascular volume status. Further studies of the compression CI of the IJ, as well as direct comparison to IVC, are warranted.

## Data Availability

The datasets generated and/or analyzed during the current study are not publicly available due to personalized/individualized data but are available from the corresponding author on reasonable request.
